# Extended Indications for Drug-Induced Sleep Endoscopy: Surgical Decision-Making in Neonatal Pierre–Robin Sequence-Associated Upper Airway Obstruction

**DOI:** 10.1055/a-2699-7977

**Published:** 2026-01-17

**Authors:** Kelvin Yong Jie Lim, Lynn Huiting Koh, Angela Yun June Tan, Gale Jue Shuang Lim, Sam J. Daniel, Ching Yee Chan

**Affiliations:** 1 Department of Otolaryngology, KK Women's and Children's Hospital, Singapore, Singapore; 2 Department of Paediatric Anaesthesia, KK Women's and Children's Hospital, Singapore, Singapore; 3 Department of Plastic, Reconstructive and Aesthetic Surgery, KK Women's and Children's Hospital, Singapore, Singapore; 4 Department of Otolaryngology – Head and Neck Surgery, McGill University, Montreal Children's Hospital, Montreal, Quebec, Canada; 5 McGill Otolaryngology Sciences Laboratory, Department of Experimental Surgery, McGill University, McGill University Health Center, Montreal, Quebec, Canada

Upper airway obstruction (UAO) is a hallmark of Pierre–Robin sequence (PRS). PRS patients are prone to obstructive sleep apnea (OSA), with severe cases experiencing airway obstruction even when awake. Severe cases often require surgical management with tongue lip adhesion (TLA), mandibular distraction osteogenesis (MDO), or tracheostomy. Tracheostomy is preferred in patients with comorbidities and multilevel obstruction, while TLA or MDO is the first line of treatment if isolated glossoptosis and/or micrognathia result in airway obstruction. There are proposed treatment algorithms, 1 2 but none so far guide the surgical decision between TLA or MDO.

Drug-induced sleep endoscopy (DISE) uses a flexible endoscope to evaluate the site, pattern, or shape, and severity of UAO in an OSA patient asleep under pharmacological means. The VOTE classification is commonly used and describes the degree (none, partial, or complete) and configuration of obstruction at the velum, oropharynx/lateral wall, tongue base, and epiglottis. 3 In a pediatric DISE expert consensus statement, 4 positional maneuvers can be performed to assess their impact on obstruction, though they are more commonly used to evaluate the effectiveness of positioning devices in adult OSA. 5 With two cases, we demonstrate that DISE can evaluate the levels of UAO in symptomatic PRS patients, and the efficacy of positional maneuvers during DISE may guide decision-making between TLA and MDO.

Our study had informed parental consent and was exempt from Institutional Review Board approval. Two patients underwent DISE for PRS-associated UAO. During DISE, the jaw was lifted upward and forward to perform a jaw thrust (JT) to simulate MDO, and the tongue was grasped with a moist gauze and pulled forward for tongue retraction (TR) to simulate TLA. The VOTE score at baseline and during each maneuver was recorded. Patient A was 2 months old and presented with severe OSA and laryngomalacia. Patient B was 4 months old with ventilator dependence due to UAO and had failed extubation twice. Tracheostomy was unsafe because of difficult surgical anatomy and limitations in patient care due to social circumstances. At rest, Patient A had partial and complete obstruction at the level of the tongue base and epiglottis, respectively. With JT, there was no obstruction at the tongue base and partial obstruction at the epiglottis, partially contributed by tight aryepiglottic folds. TR did not improve the VOTE score. Conversely, Patient B had a better improvement with TR than JT ( Fig. 1 ). At rest, there was complete obstruction at the level of the tongue base and epiglottis. With JT, the obstruction at both levels was partial. With TR, there was no obstruction at the tongue base and partial obstruction at the epiglottis level. Patient A underwent a supraglottoplasty for laryngomalacia and the OSA was managed successfully with continuous positive airway pressure. In view of a more open airway with TR, Patient B underwent TLA, was successfully extubated to bilevel positive airway pressure and discharged home ( Fig. 1 ).

**Fig. 1:**
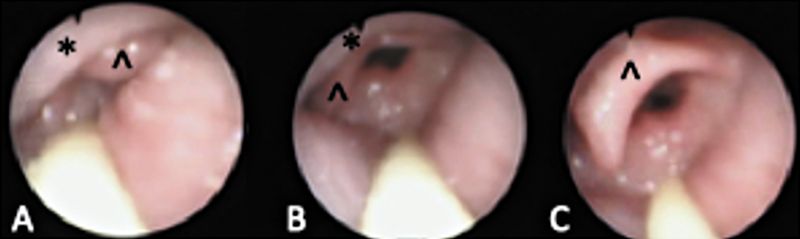
Endoscopic view of the larynx of patient B. ( **A** ) Baseline view of the supraglottis. *Tongue base, ^epiglottis. Glossoptosis causes posterior displacement of the epiglottis with complete airway obstruction. ( **B** ) Jaw thrust position, there is partial obstruction at the tongue base and epiglottis. ( **C** ) Forward tongue retraction, the airway is further improved with no obstruction at the tongue base and partial obstruction at the epiglottis.

The practice patterns of managing airway obstruction in PRS vary widely. In the nineties, before MDO became popular, TLA and tracheostomy were the main techniques. The GILLS score was described to help identify patients who would benefit from TLA. 6 Argamaso 7 also described using nasoendoscopy to confirm the presence of glossoptosis prior to performing TLA. After MDO gained popularity in the early 2000s, it replaced TLA completely in some centers. Recent data favor MDO, citing a lower conversion to tracheostomy and higher oral feeding rates. 8

PRS can happen in isolation, or as part of a syndrome. Given the phenotypic heterogeneity of PRS patients, with the exception of tracheostomy, one surgery cannot fit all. While data show MDO outcomes to be superior, some patients derive more benefit from TLA. Patient B has shown a better airway with TR than JT, favoring TLA.

While DISE is more widely performed, its use as a decision-making tool between MDO and TLA has not been described. Published algorithms either omit TLA completely, opting for MDO in isolated PRS and tracheostomy in syndromic PRS, 1 or use TLA and MDO interchangeably 2 9 with no criteria guiding the choice of procedure. Our findings suggest that DISE can contribute to the management algorithm of airway obstruction in PRS ( Fig. 2 ). To reduce interobserver variability with the VOTE classification, future directions may include corroborative measurement of oxygen saturation during each DISE maneuver to support the choice of procedure.

**Fig. 2:**
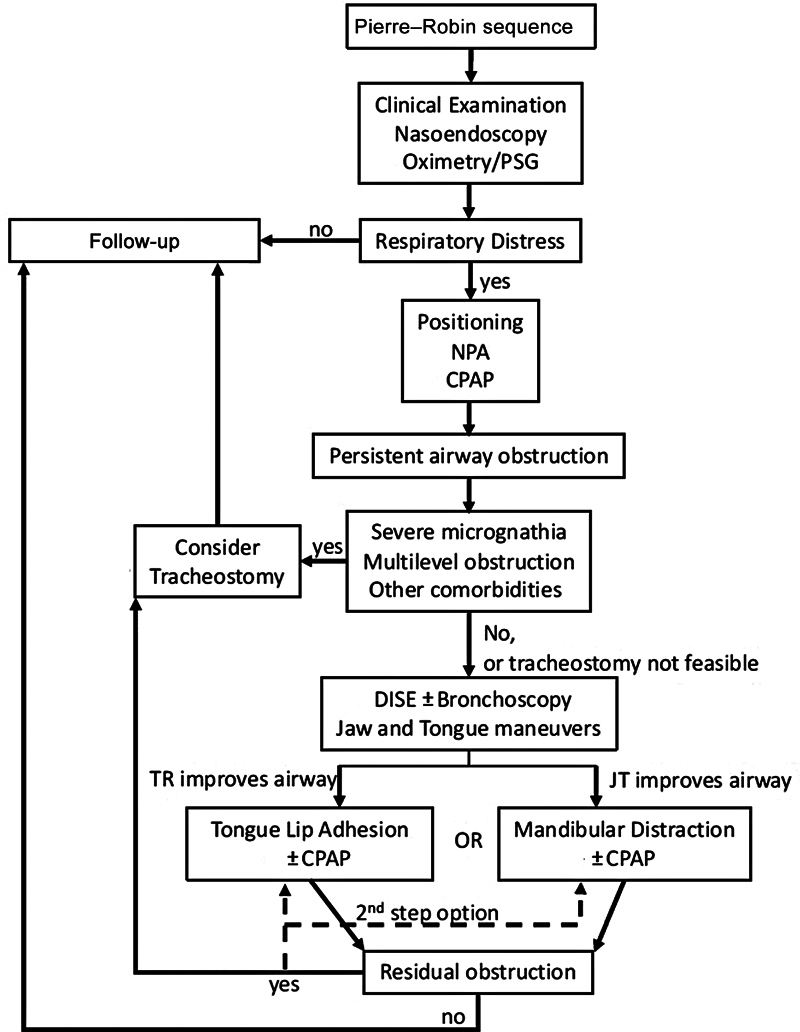
Proposed algorithm to include DISE in patients with PRS with upper airway obstruction. After initial TLA or MDO, patients with residual obstruction will either need a second surgery or tracheostomy for definitive airway. CPAP, continuous positive airway pressure; DISE, drug-induced sleep endoscopy; JT, jaw thrust; MDO, mandibular distraction osteogenesis; NPA, nasopharyngeal airway; PRS, Pierre–Robin sequence; PSG, polysomnography; TLA, tongue lip adhesion; TR, tongue retraction.

## Conclusion

We propose an extended indication for DISE to evaluate UAO in symptomatic PRS patients and assess positional maneuvers like JT and TR. DISE findings in two patients demonstrated its utility in determining airway improvement with JT or TR, guiding surgical decisions. Incorporating DISE into PRS management algorithms may provide a personalized approach to select between TLA and MDO, optimizing outcomes for these patients.
